# Differences in Learning Characteristics Between Students With High, Average, and Low Levels of Academic Procrastination: Students’ Views on Factors Influencing Their Learning

**DOI:** 10.3389/fpsyg.2018.00808

**Published:** 2018-05-28

**Authors:** Lennart Visser, Fred A. J. Korthagen, Judith Schoonenboom

**Affiliations:** ^1^Master’s Program for Teachers, Driestar Christian University for Teacher Education, Gouda, Netherlands; ^2^Social and Behavioural Sciences, Utrecht University, Utrecht, Netherlands; ^3^Department of Education, University of Vienna, Vienna, Austria

**Keywords:** academic procrastination, regulation of learning activities, differences between academic procrastinators, interview study, qualitative

## Abstract

Within the field of procrastination, much research has been conducted on factors that have an influence on academic procrastination. Less is known about how such factors may differ for various students. In addition, not much is known about differences in the process of how factors influence students’ learning and what creates differences in procrastination behavior between students with different levels of academic procrastination. In this study learning characteristics and the self-regulation behavior of three groups of students with different levels of academic procrastination were compared. The rationale behind this was that certain learning characteristics and self-regulation behaviors may play out differently in students with different levels of academic procrastination. Participants were first-year students (*N* = 22) with different levels of academic procrastination enrolled in an elementary teacher education program. The selection of the participants into three groups of students (low procrastination, *n* = 8; average procrastination, *n* = 8; high procrastination, *n* = 6) was based on their scores on a questionnaire measuring the students’ levels of academic procrastination. From semi-structured interviews, six themes emerged that describe how students in the three groups deal with factors that influence the students’ learning: degree program choice, getting started with study activities, engagement in study activities, ways of reacting to failure, view of oneself, and study results. This study shows the importance of looking at differences in how students deal with certain factors possibly negatively influencing their learning. Within the group of students with average and high levels of academic procrastination, factors influencing their learning are regularly present. These factors lead to procrastination behavior among students with high levels of academic procrastination, but this seems not the case among students with an average level of academic procrastination.

## Introduction

Many students in higher education are not successful and encounter academic failure (Vossensteyn et al., 2015). One of the factors associated with academic failure is academic procrastination (Steel, 2007; Kim and Seo, 2015), although there are also instances in which the pressure created by procrastination can improve performance (Kim and Seo, 2013). When students procrastinate, they often experience problems with learning activities, for example, with starting on time to prepare for exams and dealing with deadlines for assignments. In the present study, we define procrastination as *the voluntary delay of an intended and necessary and/or (personally) important activity, despite expecting potential negative consequences that outweigh the positive consequences of the delay* (Steel, 2007; Klingsieck, 2013, p. 26). In the context of the present study, the “important” activities are learning activities.

Various studies clarified that academic procrastination is common among college students. Schouwenburg (1992) conducted a study among 278 Dutch undergraduate college students, showing that more than 70% reported academic procrastination. About 20% reported chronic academic procrastination. In a meta-analysis, Steel (2007) cited research showing that 80–90% of undergraduate college students report that they experience procrastination in some form (Ellis and Knaus, 1977; O’Brien, 2002). Özer et al. (2009) investigated the prevalence of academic procrastination in 203 Turkish undergraduate college students and found that 52% of them reported frequent academic procrastination. Although the estimated percentages of procrastination in these studies differ, academic procrastination clearly is a serious problem and has negative consequences. Students who procrastinate regularly receive lower course grades and lower final exam grades (Wesley, 1994; Steel, 2007), and these students are less successful in their degree programs (Visser et al., 2015).

The present study examines learning characteristics of students with different levels of academic procrastination and how they deal with factors that possibly influence their learning. We conceptualize learning characteristics as those features of learning processes that students themselves indicate as relevant to their procrastination behavior, as we were interested in students’ views of factors influencing their learning.

### Factors Influencing Procrastination

Academic procrastination can be seen as a prevalent and pernicious form of self-regulatory failure (Steel, 2007). Self-regulation is a process through which the student activates and maintains thoughts, feelings, and behavior necessary for achieving personal goals (Zimmerman and Schunk, 2011). Differences in self-regulation among students contribute considerably to differences in students’ academic achievements (Zimmerman and Martinez-Pons, 1988) and levels of procrastination (Steel, 2007; Klassen et al., 2008; Grunschel et al., 2013).

In the self-regulation process, three different phases can be distinguished, when a student sets himself or herself to a given learning activity (Schunk and Ertmer, 2000; Pintrich and Zusho, 2002). The first phase is the forethought and planning phase. In this phase, the student plans his or her study activity, while various thoughts regarding motivation, values, and goals are active. The second phase is the monitoring performance and motivation phase. In this phase, the student has already started the study activity and tries to control his or her motivation and academic performance. For example, during the study activity the student can decide to change his or her learning strategy because it does not have the desired effect. The third phase is the phase after completing the study activity. This is the reflection on performance phase. During this reflection phase, the student attempts to understand why a certain result occurred and manages his or her emotions with respect to this result. In each of the self-regulation phases, procrastination problems can occur Grunschel et al. (2018). For example in the first phase, students with low levels of self-efficacy (e.g., Waschle et al., 2014) and deficient time management and goal-setting skills (Lay and Schouwenburg, 1993) have more procrastination problems. During the performance phase, procrastinating students show low perseverance (Grunschel et al., 2013) and high susceptibility to social temptations (Dewitte and Schouwenburg, 2002). In the self-reflection phase, academic procrastinators tend to make external and global attributions regardless of whether their performance was a success or not (Ferrari, 2001).

#### Personal Factors

Procrastination is influenced by several personal factors. Factors that may increase the tendency to procrastinate include anxiety (Spada et al., 2006), depression (Uzun Ozer et al., 2014), self-handicapping (*r* = 0.53; Ferrari, 2004), the Big Five factor neuroticism (*r* = 0.26; Van Eerde, 2004), fear of failure (*r* = 0.63; Ferrari, 2004), perceived competence (Haghbin et al., 2012), and pessimistic academic attributional style (*r* = 0.14; Visser et al., 2015). There are also factors that may decrease the tendency to procrastinate. These factors include self-esteem (*r* = -0.28; Van Eerde, 2003), self-efficacy (*r* = -0.54; Ferrari, 2004), self-control (*r* = -0.58; Steel, 2007), and the Big Five factors extraversion (*r* = -0.08), agreeableness (*r* = -0.10), openness (*r* = -0.15), and conscientiousness (*r* = -0.65; Van Eerde, 2004). Volitional control, social relatedness, and task competence are also important aspects influencing students’ procrastination (Klingsieck et al., 2013).

When students have to do a learning activity for their degree program, it is important that they have a certain level of the executive functioning domains of initiation, plan/organize, organization of materials, and task monitoring (Rabin et al., 2011). There is a higher risk of academic procrastination if students have poor planning skills (Rabin et al., 2011), a reduced use of cognitive and meta-cognitive learning strategies (Howell and Watson, 2007), or a low level of perseverance, and if the students are easily distracted (Dewitte and Schouwenburg, 2002). It is also important that students are motivated for the learning activity (Grunschel et al., 2013). Motivation is the force that drives a person to engage in activities (Amabile, 1983) and concerns energy, direction, persistence, and all aspects of activation and intention (Ryan and Deci, 2000). Student motivation can be distinguished (Ryan and Deci, 2000) as intrinsic motivation, that is, motivation resulting from internal drives (doing a learning activity for the pleasure it brings or because of interest) and extrinsic motivation, that is, motivation resulting from external factors (doing something for an external reason; for example, the student wants to maintain a good relationship with the teacher). High intrinsic motivation reduces academic procrastination (Lee, 2005; Steel, 2007). Students have a mastery-approach goal orientation (Howell and Watson, 2007) when they focus on learning, mastering the task according to self-set standards, or when they improve themselves and develop new competencies. Mastery goal orientation is an important factor that prevents academic procrastination (Seo, 2009). When a learning activity does not intrinsically motivate a student, he or she has to actively transform external regulation into internal regulation and shift from external control of his or her behavior to internal control of his or her behavior. This is the so-called process of internalization (Ryan and Deci, 2000).

#### Situational Factors

Academic procrastination can also be influenced by situational factors. An important situational factor is teachers, because if teachers are well-organized, it is easier for students to organize, structure, and plan their work (Corkin et al., 2014). Unorganized and lax teachers can be a reason for students’ procrastination (Grunschel et al., 2013). Procrastination is also promoted by teachers when they expect less, are willing to negotiate deadlines, and are more flexible in their grading (Schraw et al., 2007). Teachers with high expectations increase students’ class enjoyment and interest and diminish students’ procrastination (Corkin et al., 2014). When a teacher provides clear instructions for assignments, students procrastinate less (Ackerman and Gross, 2005).

When students have to do study tasks, procrastination can be evoked and maintained by task characteristics (Nordby et al., 2017). A task perceived as boring, unpleasant, and/or uninteresting (so-called task aversiveness) strongly predicts students’ procrastination (Blunt and Pychyl, 2000; Steel, 2007). At the start of a project, task aversiveness is related to personal meaning, such as pleasure, fun, enjoyment, and communion. When a task is perceived as interesting or requires students to use a variety of skills, and when students perceive social norms and rewards for starting promptly, students procrastinate less (Ackerman and Gross, 2005). An optimal degree of task difficulty is reached if a task is sufficiently challenging but also achievable (Van Eerde, 2003; Steel, 2007).

### Differences in How Students Deal With Procrastination

Thus, various factors could influence students’ procrastination behavior. However, there also can be differences between students in how they are influenced by these factors. Interviews with experienced university counselors showed that antecedents and consequences of academic procrastination are largely a reflection of students’ characteristics, personal and learning situations, and the environment of the university (Patrzek et al., 2012). There can also be differences between students’ delaying behavior and having academic procrastination, because delaying a task can be an intentional decision. For example, students intentionally delay because of other priorities or because they feel they can work better when they set aside a designated period of time (Lindt et al., 2014). Grunschel et al. (2013) showed that students who are pressure-seeking types are not negatively affected by academic delays and have low academic procrastination.

An interesting study for insights into differences between students with different levels of academic procrastination was conducted by Nordby et al. (2017). Their study showed that differences in students’ academic procrastination are associated with the manner in which students deal with environmental factors that might provoke procrastination behavior. Academic procrastination (in the form of choosing social activities when intending to do academic work) and the evaluations of other students’ procrastination habits were higher in students in the field of humanities compared to the field of natural sciences and medicine. Differences between students were moderated by the dispositional tendency to procrastinate. The humanities students demonstrated a higher level of socially induced academic procrastination and evaluated other students’ procrastination habits (peer procrastination) more than students in medicine and natural sciences did. Environmental factors had a negligible impact on academic procrastination on students with a low disposition to procrastinate. For students with medium levels of dispositional procrastination, which are the majority, procrastination-friendly environments facilitate and augment the students’ procrastination.

### Research Gap

Academic procrastination is a highly complex human behavior that involves a combination of affective, cognitive, and behavioral components (Brownlow and Reasinger, 2000; Chun Chu and Choi, 2005). Despite the extensive, mainly quantitative, research concerning procrastination, there is still a lack of understanding of why students procrastinate (Steel, 2007; Klingsieck et al., 2013; Katz et al., 2014).

Because we wanted to get insights into differences among students with high, average, and low procrastination, we selected students with different measured levels of procrastination. This is an important difference compared with previous qualitative studies on academic procrastination. Most previous qualitative studies about academic procrastination (e.g., Grunschel et al., 2013; Klingsieck et al., 2013; Lindt et al., 2014) included students with high academic procrastination. The selection of the respondents in those studies was not based on students’ measured levels of academic procrastination, but students were, for example, approached on the university campus restaurant by the interviewers and asked whether they would be willing to participate in an interview study concerning academic procrastination (Klingsieck et al., 2013). In other studies, flyers were distributed on the university campus with a call to participate in the study (Grunschel et al., 2013), or students were asked to fill out a questionnaire between lectures or students were contacted through the course forum when they did not attend lectures (Nordby et al., 2017).

### The Present Study

Therefore, we know that factors influencing procrastination can work differently among students from different academic disciplines and among students with different procrastination levels (Nordby et al., 2017). We do not know how the process behind these differences works. In the present study, we wanted to dig deeper into the process behind students’ academic procrastination and how students deal with factors that might influence their learning. We see the present study as an important continuation of Nordby et al.’s (2017) study in which they concluded that social and environmental factors should receive increased attention and suggested that future studies should include more diverse measures. We conducted the present study from a qualitative perspective, because students’ views (Klingsieck et al., 2013) could show more about their learning characteristics whether and how they deal with procrastination. With this study, we wanted to provide new insights for theoretical groundwork in procrastination research and provide insights that can be used for developing interventions that teach students how to handle their procrastination. This is important for educational institutions and people in these institutions who work with students, such as teachers, counselors, and educational psychologists.

### Research Question

The following research question guided the present study: what are differences between students with low, average, and high levels of academic procrastination in learning characteristics and in factors that might influence students’ learning?

## Materials and Methods

To our knowledge, the present study is the first study to explore differences in learning characteristics and in the influential factors on academic procrastination among students with low, average, or high academic procrastination tendencies. We conducted a qualitative interview study because we aimed to explore this topic without preliminary hypotheses and wanted to get insight into students’ actual experiences and regulation of learning activities, and how they relate to the students’ study practices.

### Sampling

In this study, we interviewed 22 students (7 men, 15 women, age between 16 and 22 years; *M* = 17.7, *SD* = 1.4). All students were freshmen in a full-time 4-year elementary teacher education program at a small teachers college with approximately 1,500 students, situated in the western region of The Netherlands. To select the participants for the interviews, we first wanted to know at what level students actually experienced academic state procrastination in the week before their exam period. Therefore, we measured the students’ academic procrastination with the Academic Procrastination State Inventory (APSI; Schouwenburg, 1995, unpublished). The APSI is an existing Dutch questionnaire that meets the requirements of internal reliability and validity (α = 0.94) and has been used in previous research (e.g., Höcker et al., 2008). In the APSI, the student is asked about his or her study behavior during the week before completing the questionnaire. Each of the 31 items begins with the question “How often did you… last week?” On a five-point Likert scale from never (1) to always (5), the student indicates his or her assessment of how often something happened.

After measuring all first-year students’ (*N* = 215) levels of academic state procrastination, we used the data of the students who completed the APSI questionnaire (*N* = 186; 26 men, 160 women, age between 16 and 22 years; *M* age = 17.62, *SD* age = 1.03; *M* APSI = 81.68, *SD* APSI = 19.38, *α* APSI = 0.93). We ranked the outcomes and classified three groups of students, based on their APSI scores: students with the lowest sum score, students with an average sum score, and students with the highest sum score for academic state procrastination. In the order of the sum score ranking (beginning with the lowest-scoring student of the low procrastinators, the average-scoring student of the average procrastinators, and the highest-scoring student of the high procrastinators), the first author of this article approached students personally. He informed them about this study and asked whether they were willing to participate in the study and to be interviewed about how they gave meaning to their learning activities. As a result, 22 students were interviewed: eight students with low procrastination scores, eight students with average procrastination scores, and six students with high procrastination scores. See **Table 1** for the APSI scores of all first-year students and the groups of selected participants.

**Table 1:** Academic Procrastination State Inventory (APSI) scores of the selected participants and all first-year students.

	Age				Academic state procrastination^∗1^			
		
	Minimum	Maximum	Mean	SD	Minimum	Maximum	Mean	SD
***Low procrastinators***	16	22	17.75	1.83	36	56	49.13	6.15
*N* = 8: three males, five females		
***Average procrastinators***	16	20	17.75	1.28	73	81	78.89	2.75
*N* = 8: two males, six females		
***High procrastinators***	17	19	17.50	0.84	110	129^∗2^	117.00	7.01
*N* = 6: two males, four females		
***All first-year students***	16	22	17.62	1.03	36	131	81.68	19.38
*N* = 186: 26 males, 160 females		


*^∗*1*^The lowest possible minimum score is 31; the highest possible maximum score is 155. ^∗*2*^The APSI maximum of the high procrastinators differs from the APSI maximum of all first-year students, because not all high procrastinators we approached were willing to be interviewed.*

### Interviews

All the interviews were conducted individually by the first author of this article. The interviewer was not familiar with the students and had no relationship with them. At the start of the interview, the interviewer gave information about the interview process. He explained that the interview would take about 45 min and that the interview was about how the student gave meaning to his/her learning activities. The interviewer explained that he wished to make an audio recording and asked the student for permission for this. He stated that the interview would be processed anonymously and that the conversation was an open conversation, in which there would be no right or wrong answers.

The interview protocol for this study was structured by seven general questions. The interviewer used the questions as guidance but also probed specific issues that came up during the interview. The interview questions were general questions regarding students’ personal experiences with preparing for assignments and exams, as well as questions to better understand students’ procrastinating processes and the influential factors (see **Table 2**). With the interview questions, we wanted to cover the three phases of the self-regulation process (Schunk and Ertmer, 2000; Pintrich and Zusho, 2002): the forethought and planning phase, the monitoring performance and motivation phase, and the phase after completing the study activity, as well as factors influencing the students’ learning. During the interviews, the interviewer used the protocol to check whether all interview questions were covered. On average, the interviews lasted 46 min (*SD* = 9).

**Table 2:** Overview of the interview questions.

Interview questions	
(1)	Why did you choose this degree program?
(2)	If you have to study/complete assignments for your study, how do you get started? Can you describe your approach?
(3)	How is it for you to be engaged in study activities?
(4)	How is it for you if, while performing study activities, you realize that it is going well? What do you think? What do you say to yourself?
(5)	How is it for you if, while performing study activities, you realize that it is not going well? What do you think? What do you say to yourself?
(6)	How do you appreciate yourself in general?
(7)	What expectations do you have about the results, before an examination? Do you expect them to be positive or negative? What explanation do you have for that result?


The procedures of this study were carried out in accordance with the recommendations of the local guidelines of the Faculty of Behavioural and Movement Sciences, VU University Amsterdam (Vrije Universiteit Amsterdam, 2016). This study was approved on these guidelines by the local ethic committee Research Centre of Driestar Christian University. All subjects gave written informed consent in accordance with the Declaration of Helsinki.

### Analysis

The interviews were anonymously transcribed by a research assistant. The first author read the interviews to get an overall impression and then segmented the text into meaningful units that were comprehensive by themselves and contained one idea, episode, or piece of information (Tesch, 1990; Schilling, 2006). The first author coded nine selectively chosen interview transcripts (the highest-, middle-, and lowest-scoring participants of each subgroup of procrastinators) and composed the codebook, consisting of 18 codes. For the coding procedure, the software ATLAS.ti was used. To calculate intercoder reliability, a research assistant independently coded the nine interviews. Cohen’s kappa was calculated for the nine interviews, based on the 18 codes and the 193 segments. The intercoder reliability was good (*κ* = 0.82). After this procedure, the first author coded the remainder of the segments (13 interviews, consisting of 324 segments).

To further structure the data, we performed a qualitative content analysis using a thematic analysis approach (Fereday and Muir-Cochrane, 2006) to identify overarching themes that captured the differences in learning characteristics and the factors that influence students’ learning, as described by participants in the interviews. This analysis resulted in six main themes. To promote validity, the authors applied peer debriefing several times until consensus was reached, and they critically discussed the results of the coding process, the formulation of overarching themes, and the selection of the illustrative quotes. When one or more participants gave answers that contradicted one of the themes we described, we did not neglect these quotes but used them to further elaborate the findings and mentioned the quotes in Section “Results.” We also counted how often quotes occurred in relation to the total number of the students in a subgroup and showed this in Section “Results.” See **Table 3** for an overview of the codes and themes.

**Table 3:** Overview of codes and themes.

Codes	Themes	Explanation
Motivation to be a teacher	(1) Degree program choice	Student’s motivation for his or her degree program choice
Approach when performing study activities	(2) Getting started with study activities	Student’s approach for getting started with study activities
Positive feeling(s) when working on or completing study activities	(3) Engagement in study activities	How the student is engaged in study activities
Being in the flow: absorbed in the study activity		
Motivation enhancing the study experience or study activity		
Postponement behavior	(4) Ways of reacting to failure	How the student reacts to failure or difficult moments during study activities
Distracting thoughts during study activities		
Focus on what is finished or what still needs to be done		
Need for fun and contact with friends or other people		
Dealing with setbacks when the activity is not successful		
Dealing with moments of lack of motivation/boring study tasks/not seeing the usefulness of the study activity		
Dealing with distracting multimedia (Facebook/WhatsApp)		
Sense of conscience/discipline/responsibility		
Attitude of life		
Belief in oneself or in one’s ability to succeed	(5) View of oneself	The way the student sees himself or herself
Attitude of acceptance	(6) Study results	Expectations for and evaluations of study results
Expectation about exam results/results of assignments/feelings of anxiety about exams		


## Results

Six themes emerged from the interview data, which we discuss below. In these six themes, there were clear differences between all three groups of students, or between one group and the other two groups. In the description of each theme, we present between brackets (…/…) how often quotes occurred in relation to the total number of the students in that subgroup. Every theme is illustrated with a representative quote. When one or more students in the subgroup gave answers related to the theme that contradicted the quotes we described, we also give representative examples of these contradictory quotes. The student names cited for each quote are pseudonyms. See **Table 4** for a summary of the results.

**Table 4:** Summary of the results.

	Low procrastination students	Average procrastination students	High procrastination students
Degree program choice	Intrinsically motivated decision to become a teacher.	Intrinsically motivated decision to become a teacher.	Have no clear idea of becoming a teacher.
	Choice confirmed by positive experiences with theoretical and practical parts of the program.	Internships are important experiences motivating students to continue with their program when they have doubts about it.	Have doubts about the program and consider quitting.
Getting started with study activities	First focus on the description of the activities content, nature of the material, and assignments requirements, and then plan tasks and goals to achieve.	First focus on content or number of activities and then plan tasks and goals to achieve.	Plan learning activities, but carrying out the plan depends on certain preconditions.
	Set no preconditions to start.	Set no preconditions to start.	Set preconditions to start. If preconditions are not met, the learning activity will be postponed.
Engagement in study activities	Intrinsically motivated and go for it.	Focus on completing study activities and less so on possible takeaways.	Focus on the utility of the study activity. When the learning activity appears to be useful and enjoyable, they enjoy doing it.
	Aware of how the activity is going and the progress they make. Consciousness of gaining insights and general knowledge.	Progress of the task is determined by relevance to the profession and applicability during internships. If not, it’s hard to remain engaged.	When a learning activity is boring and considered stupid, they tend to stop doing it, turning to other non-school activities that are more appealing.
	Further their knowledge.	Reflect on own role as a teacher when learning activity bears on the profession.	
Way of reacting to failure	Remain focused on completing the learning activity when the result is not desirable.	Do less and think they are wasting their time when the result of a learning activity is not desirable.	When progress is disappointing, then judge themselves negatively.
	Encourage themselves verbally to keep going.	Experience a sense of failure and feel low or moody.	Experience negative feelings and low self-esteem.
	Rely on their capacities to complete the learning activity.	Are hopeful that they will manage and expect that, in the end, they will be able to complete.	No longer believe that they are up to it. Those negative feelings can also concern situations outside the program
View of oneself	Satisfied with the person they are.	In general satisfied with the person they are, but also critically reflect on themselves seeing points for improvement.	Some moments of not being satisfied with the person they are. The esteem others have for them is also important.
	Positive self-esteem.	Positive self-esteem.	Frequent moments of negative self-esteem.
Study results	Confident about results beforehand, which is reinforced by good results in the past.	Sometimes doubtful beforehand about the results.	Confident beforehand if they know they spend enough time to prepare for exams.
	Levelheadedness, no stress or nerves for exams.	Nervous about exams and feel pressure to do well.	No fear or nerves about exams.
	Passing the exams is explained by their own efforts.	Expectations of passing or failing depend on the difficulty of subject.	See themselves as the determining factor in passing or not. If they fail, they attribute their failure to not spending enough time preparing for the exam and/or to not attending all lectures.


### Theme 1: Degree Program Choice: Intrinsically Motivated Versus “I’d Rather Do Something Different”

#### Students With Low Levels of Procrastination

When considering statements about motivation for doing the degree made by the group of students with low levels of procrastination (“LP students”), all LP students reported this choice was intrinsically motivated, as the logical way to fulfill their dream of becoming a teacher (8/8).

I am highly motivated to do this degree. It simply is my dream to become a teacher (…). (Tiffany)

All the LP students were intrinsically motivated for the degree they were doing. They described it was the right choice which was confirmed by their positive experiences with the theoretical and practical parts of the program (8/8).

The assignments are so interesting and fun to carry out that you really enjoy doing them. You get a positive energy to carry on. This feeling—I can do it, I’ve submitted it, it looks good. And then, “Okay, up to the next one…” It’s just nice to experience it like this. (Lucas)

Most of the LP students felt it was self-evident that they should be committed to the program and persevere. They felt they were accountable for the choice they made for their degree (5/8).

It is simply perseverance, because I want to. I just want to get those credits and do well. I really think it is important. You decide to take this degree, so you really go for it…. My approach is purposeful. I have a goal in mind, and I feel responsible for it. Because I chose to do this, it is up to me to do well. I want to become a good teacher, so it’s important to go to class and make sure you learn from it. (Irma)

#### Students With Average Levels of Procrastination

All students (8/8) with average levels of procrastination (“AP students”) stated they carefully chose their degree program. They had a clear intrinsically motivated goal in mind for doing the degree; they wanted to become a teacher.

I’ve known for a long time, since I was four years old, that this is what I want. So I think like, this is your drive to get things done after all. (Marvin)

Most AP students reported they were more focused on the practical part and less on the theoretical part of the degree (6/8). The internships in the field of their profession were very important to most AP students (7/8). When AP students had doubts about their degree program choice, most drew energy from their internship experiences, which provided them with the incentive to continue their degree (5/8). They realized once again why they wanted to do this, namely, to become a teacher.

With some assignments, I have that kind of feeling like, what for? Surely, I don’t need all that to become a teacher…. Doing the internship confirms my choice, I really enjoy doing that. But at school I don’t always have that feeling. I just love the practical work. So then I think, well I’ve just got to get through those four years. The internship makes that I realize that this is what I really want. (Ailyn)

#### Students With High Levels of Procrastination

Students with high levels of procrastination (“HP students”) described they did not carefully choose their degree program. Finishing the degree or becoming an elementary school teacher was not the primary intrinsically motivated goal for the majority of HP students (4/6). Most of the students reported their interests lay elsewhere (4/6). The students had a wide range of reasons for still opting for an elementary teacher education program.

I didn’t have that feeling like “this is what I want later in life” when I opted for this degree. Of course, if you think this is super fun, you will be much more dedicated to the program. But that hasn’t been the case for me from the start. I’m here because I didn’t have a better idea…. Once I’m done here, I should like to continue at university, I’m kind of interested in History. (Jack)

Most HP students (5/6) stated they were doubtful about continuing their degree and considered quitting. Some (3/5) of these HP students who were doubtful about continuing their degree and considered quitting their studies, explained that the program content and the learning activities that came with it had little appeal.

If all day long all you do is being busy with those stupid assignments, you do start to wonder, why am I doing a degree in elementary teacher education? I’d rather do fun things. I’ve got that quite often…. So I’m not really that positive about the program. I keep wondering whether this is the right choice for me. (Jennifer)

For some HP students (2/6) who carefully chose their degree program, the way things were going at the college gave them the feeling they were not in the right place. Therefore, they wanted to continue their studies at a different teacher training college.

This college just isn’t the place for me. I find it too strictly Christian…. I’ve enrolled in a different college for next year. (Ariah)

### Theme 2: Getting Started With Learning Activities: Just Get Started Versus Get Started Only Under Certain Preconditions

#### Students With Low Levels of Procrastination

Most of the LP students (6/8) stated, when they had to perform learning activities for the program, their approach was to focus first on the description of the content of the activity. In this way, they assessed the nature of the material and the assignment’s requirements. Based on this orientation, they planned the different tasks, considering the goals they wanted to achieve for completing the activity. Then the students performed the learning activity. They did not set any preconditions for getting started.

The first thing I do primarily is to look at the assignment, see what it says. Its requirements, what I need to do. Then I start with the assignment until I’m done. Or at least I get started with it. I see how far I get, how much time I’ve got…. I make a to-do list for the assignments. (Audree)

All LP students (8/8) stated, that when they started with their learning activity, they found out easily whether they had made the correct assumptions about it, and then they expected to see how far they get.

I primarily make sure I understand the assignment and if I don’t, just ask. And then I just get started with it and see how far I get. If I’m stuck, I just see what I can do about it so that I can get on with it. You just need to know what’s expected… I could start straightaway, but then it would go like, I’m in the middle of it, and I realize this should go in and that as well and I shouldn’t have done such and such because I don’t need it at all. And I don’t like that! (Lucas)

#### Students With Average Levels of Procrastination

Most of the AP students (7/8), described that, when they had to perform learning activities for the program, they did not set any preconditions to get started and just begin. Four of these AP students who did not set any preconditions planned their work after orienting themselves to the content of the activity. The other three planned their work after it was clear how much they needed to do. Their study behavior was guided by making themselves familiar with the demands of the assignment and its deadline.

At the start of the term, I read the course guides and note down the assignments I have to do for each course. I put them in a schedule, so that I know, this is what I have to do in this term and those are the deadlines. It’s a kind of goal I set myself. I find it quite useful…. So that I have an absolutely clear overview of what needs to be done in terms of assignments and exams. (Juliet)

#### Students With High Levels of Procrastination

When HP students had to perform learning activities for the program, most reported they made a study plan (5/6). Whether the plan was actually executed depended for most of the HP students on certain preconditions they have (5/6). Examples of such preconditions regarding study activities are: does the activity yield enough credits? Is the activity attractive and fun to do? Is it clear what must be done? Is the activity not too difficult? How nearby or far is the deadline when the activity must be done? Does the activity concern group work? Some conditions have to do with the student, such as they need a clear overview in order to get started or must feel some enthusiasm for it, be in the right mood for it, or be confident about their ability to complete the learning activity successfully.

I’m not good at planning my work. I can make a plan but I do not stick to it. I kind of think, what appeals to me now, or more often really, what must be done now or is there any group work that has priority, because with group work you must do your bit. With the other stuff I look at how important it is, how soon I’ll get credits for it, things like that. (Jennifer)

In the absence of preconditions, most of the HP students described they easily postponed learning activities (4/6).

If an assignment isn’t clear, and I believe it is difficult, I postpone it. Because I feel that ‘I won’t be able to do it anyway,’ or ‘I don’t know how to go about it.’ (Ariah)

Most HP students told they often thought they had enough time to do the learning activity and postponed getting started, thinking “there’s still time” (4/6). Such procrastination behavior could occur at different moments: at the start of the term when there was still enough time, but also when the deadline was near, such as the week before the submission of a report or even the evening before the day of an exam.

If I read the summary half an hour before it, I usually get a pass. For example, I think, I’ve got an hour, so if I get started at nine o’clock, I’ll get it done. And then it is nine thirty, and I think, oh well, tomorrow is another day. (Jennifer)

### Theme 3: Engagement in Learning Activities: Interested in the Content for Its Own Sake Versus Usefulness of Content in Practice

#### Students With Low Levels of Procrastination

Reporting about performing learning activities, most LP students (7/8) told they were conscious of their own commitment to the activity and of how the activity was going. When they (5/8) found the content interesting, they looked to further their knowledge. Because they were interested in the content, they actively became more engaged, deepening their knowledge and doing more than was required by the assignment.

If I’m interested, I learn from it. What those people say, I then think, hey, that’s a good one, I want to find out more about this. I then sometimes search for articles in newspapers or for discussions about it. (Tiffany)

In the experience of LP students, performing learning activities was something they enjoyed (5/8). They were aware that they were gaining important skills and knowledge from doing these activities and that these skills contributed to their personal development.

Looking at how far I’ve come and what I’ve learned. It all contributes. General knowledge. Knowledge about teaching, insight into yourself. Really very useful. (Lucas)

Whether certain learning activities did not look interesting or useful to LP students, most students still continued doing them (7/8). The students described they realized that completing the unenjoyable learning activity was necessary to be able to complete their degree. In their minds, the activity was just something that formed part of the curriculum and just has to be done. They showed perseverance, wanting to get it done.

I do not always see the usefulness of an assignment. You’ve still got to do it though, to build your knowledge and be able to do it in practice. This is after all what’s expected of you, it’s part of your professional attitude. (Brandon)

#### Students With Average Levels of Procrastination

When AP students reported about performing learning activities, most described they focused on completing the activities (5/8). These students were less interested in the question of what they would take away from it. Most AP students were motivated to do the learning activity if the activity looked inherently interesting (5/8). Half of the AP students reported it was easier to get started and complete the activity when they considered it interesting (4/8). In order to stay engaged with the activity when completing it, it was important to half of the AP students that the activity was interesting and/or fun. If that was the case, they felt it was easier to perform the activity (4/8).

Theories are often too remote. If I don’t find it interesting, I think, stupid assignment, I’ll leave it till later. With practical assignments, I really put myself to it, thinking, ‘I’ll make sure it’s good.’ (Amber)

Most AP students described they were interested in the learning activity if it was relevant for, and applicable to, the profession (6/8). They asked themselves, “What can or should I do with this as a teacher?” If a learning activity’s usefulness was unclear, then it had little appeal, which made starting the activity harder.

It’s more fun to do if the material is interesting and practice-oriented. Seeing how you could apply it easily makes it more appealing. It is also easier to remember. (Ailyn)

When a learning activity was not interesting or appealing, for all AP students it was harder to get started with it and persist (8/8).

When studying for my exams, I notice I’m not really into that. So I think, tomorrow is another day. Then I postpone it again. (Marvin)

#### Students With High Levels of Procrastination

All of the HP students reported it was important for them to perceive the learning activity as useful and enjoyable. In that case, they were motivated to perform it, and experienced it as pleasant to do (6/6).

If teachers would just explain the assignments in class, tell you which to do, and about their purpose. But they never do so. Why so many assignments? Just set a few small assignments with a clear purpose. That would be a huge difference for me. Then I’d do them. (Gordon)

Most HP students (5/6) described they did not perceive the utility of a learning activity if they considered it boring. They tended to stop doing it and turned to other activities that were more appealing.

If all day long all you must do is those stupid assignments. You do start to wonder, “Why am I taking this degree? What am I doing here? I would much rather do something more fun.” That happens quite often with me. (Jennifer)

### Theme 4: Ways of Reacting to Failure: Perseverance and Getting It Done Versus Doubting One’s Ability and Giving Up

#### Students With Low Levels of Procrastination

Most LP students (7/8) reported they did not give up when their efforts for the learning activity did not seem to lead to a desirable result. LP students were aware that the learning activity was not going as well as they would have liked. They were annoyed but still focused on their goal, wanting to complete the learning activity. Most LP students maintained a positive attitude, relying on their ability to eventually complete the learning activity (5/8).

If it isn’t going well, you often feel kind of, “Come on—just get on with it and then you’re done. Just carry on and then it is finished.” That’s how it works for me. It’s like “it’s something that simply has to be done, just go for it now so that it won’t bother you later.” (Whitney)

#### Students With Average Levels of Procrastination

Most AP students (7/8) described they noticed that an activity was not going well and that they were doing less than they would have liked to, when their efforts for the learning activity did not seem to lead to a desirable result. If it was not going according to their wishes, most AP students (6/8) reported they got negative feelings, thinking they were wasting their time. For example, they felt low and/or moody, or experienced a sense of failure.

But if I’m working on it and not managing, I’ll feel very negative. Thinking “Oh it isn’t going well at all, I’m just wasting my time.” I kind of drift off … And then I feel quite low. I feel a bit of a loser. (Amber)

In such situations, when performing the learning activity was not going well, most AP students (6/8) were still hopeful that eventually they would manage, expecting that in the end they would be able to complete the activity satisfactorily.

I kind of think, ‘Come on, you can do this.’ I then keep telling myself ‘just get it done, put yourself to it, and when it is finished you are done.’ (Juliet)

#### Students With High Levels of Procrastination

When the efforts of HP students for the learning activity did not seem to lead to a desirable result, these students described they judged themselves negatively (5/6). Their self-esteem seemed to drop, and they no longer believed that they were up to the task. They no longer wanted to persist, could not recover from a downward spiral, and gave up (5/6).

So then I think, “I’m stuck.” Or when an assignment is returned for rewriting for the third time, I don’t even bother. You never get it right. That wears me out. You try, make an effort, but still it’s not right. And then you think, “Why bother…” (Jack)

Most HP students reported those negative feelings also concerned situations outside the program (5/6).

Then I just think I can no longer do it. And if that happens, I have the same feeling with other things, too. I lose interest, no longer feel like it. It doesn’t have to be like that, but at such moments everything looks negative. Your self-esteem really drops. (Gordon)

### Theme 5: View of Oneself: Positive Versus Negative

#### Students With Low Levels of Procrastination

All LP students described a positive view of themselves. Their self-esteem was positive and unwavering (8/8). They knew they had the capacity to complete their degree, which was confirmed by the high grades they received.

My self-esteem is quite positive. At a certain point, you know you can do it… With negative self-esteem, thinking you aren’t up to it, I think it soon becomes harder to do… To me, it isn’t surprising really that I pass everything. My grades are quite good actually. (Audree)

Most LP students reported they were satisfied with the person they were. They accepted themselves as they were (6/8).

I am who I am. I’m okay with that. (Brandon)

No complaints. I am who I am and accept who I am. (Lucas)

#### Students With Average Levels of Procrastination

Most AP students reported they were generally satisfied with the person they were, and their self-esteem was positive (7/8). In addition, they reflected critically on themselves, seeing certain points for improvement. (7/8).

I’m happy with the person I am in most respects. Besides, there isn’t much you can change really. I’d like to be a little more outgoing with respect to other people. A little more forthcoming in groups. That doesn’t happen so often. (Julisa)

#### Students With High Levels of Procrastination

Most of the HP students (4/6) described they had frequent moments of low self-esteem.

Sometimes I think, “Ugh, I don’t want to look the mirror.” Without cause. I don’t know. How I feel also has to do with how I look. After experiencing success or when we’re having fun together, I think, hey, I don’t look so bad, and I’m okay. But on a day when I fail something, it affects everything. It’s all or nothing with me. (Ariah)

In judging themselves, half of the HP students (3/6) reported they did not consider only the way they viewed themselves but also how they were appreciated by others. For two of them this view included HP students’ view of fellow students or and for one students the view of teachers.

What someone else thinks of me is almost more important than what I think myself—that doesn’t count really, it’s not important. I don’t dare relying on my own opinion of myself. (Ariah)

### Theme 6: Study Results: “I Passed Because I Am Able To” Versus “I Failed Because of Poor Preparation”

#### Students With Low Levels of Procrastination

Most (7/8) of the LP students described they were confident about the results and embarked on the exam period with a certain levelheadedness. None of the LP students (8/8) suffered from stress or nerves.

If I know ‘I did my best,” then that’s what it is. I’m never nervous beforehand. (Brandon)

When LP students (4/6) passed an exam, most reported they believed it was due to their own efforts, because while preparing they did what they had to do.

Beforehand, I expect to pass. I simply go for it. I prepare as best as I can… I do what I can, and then it’s just a matter of wait and see. (Lucas)

Most of the LP students (5/8) described their confidence about passing their exams was reinforced by good results in the past. Previous good grades were a confirmation of the students’ abilities, boosting their confidence they would pass their exams.

Now, when facing an exam, I expect to pass. In fact, I’ve never failed an exam. (Teresia)

#### Students With Average Levels of Procrastination

Most AP students (5/8) reported they were doubtful about their exam results when they embarked on the exam period. For three of them who were doubtful, this doubt depended on the subject.

I know that in most cases I’ll pass. Sometimes, I’m afraid to fail, because it’s difficult. I’m not really good at arithmetic, for example. (Joyce)

Several AP students described their confidence in passing their exams was negatively affected by thoughts of expecting to fail (3/8).

I often have negative thoughts like ‘I won’t pass anyway.’ (Rebeca)

For two AP students, expecting to fail seemed to act as a form of self-protection (2/8). In their view, it was better to say they would fail than state beforehand that they would pass. When they failed, it was a drawback and a disappointment; when they passed, it was something of a windfall.

It’s often like, “I don’t know.” Not often like “don’t worry, you’ll pass.” That would be a little scary. In case you fail, and you thought “surely you’ll pass.” Then if you don’t pass, you’re really very disappointed…. If you depart from the notion that you’ll fail, then it could always be better than expected, right? (Juliet)

Some AP students (3/8) reported they were nervous before the exam period. This feeling went with the thought that it was all or nothing during exam week. They felt pressured to perform well.

I’m quite nervous always. I’m not sure about myself. Will I manage? The exam period is very demanding, and you have to show your best abilities. (Howard)

#### Students With High Levels of Procrastination

Most HP students (4/6) described they did not suffer from fear or nerves before exams. They saw themselves as the determining factor in passing or not passing their exams.

When I’m well prepared, I expect to pass. (Melani)

In practice, however, most HP students (4/6) reported they often prepared poorly. They did not spend enough time preparing for the exam and/or did not attend all lectures. In the students’ view, this behavior explained why they failed some of their exams.

You do hope to pass, but occasionally, you’ve got to admit that it’s not going to happen. I don’t always prepare that well, and I do tend to skip lectures, and then you miss out on quite a lot. (Jack)

## Discussion

We know that different factors might influence procrastination. The importance of this study is that it shows that there are differences between students with low, average, and high levels of academic procrastination, in whether or how factors influencing procrastination play out in practice, and whether or how this influences students’ learning.

The results show that for low and average procrastinators their self-chosen goal of becoming a teacher works as a strong intrinsically motivational drive to work on study activities and finish them. This seems to be related to what is called the need for autonomy in Self-Determination Theory (Ryan and Deci, 2000). High procrastinators lack the intrinsic motivation to become a teacher, and starting and/or continuing study activities is a problem for them. These students seem unable to externally regulate (Ryan and Deci, 2000) their behavior and are unable to shift from an external control of their behavior to an internal control of their behavior. The results are in line with Wolters (2003) who showed that the level of procrastination reported by undergraduate students can be viewed as a function of the motivational beliefs important for self-regulated learning. The results are also in line with other studies showing the role of motivation in students’ academic procrastination. For example, Grunschel et al. (2016) found that the use of motivational regulation strategies had significant positive indirect effects on students’ academic performance and affective/cognitive well-being, via academic procrastination. Brownlow and Reasinger (2000) showed that procrastination is less likely to occur for intrinsically motivated activities and Visser et al. (2015) showed that students’ lack of motivation influences their levels of academic procrastination. The motivational problems of high procrastinators can be explained with the temporal motivation theory (TMT; Steel and König, 2006). Because high procrastinators do not have a clear intrinsically motivated goal, they do not have an expectation of receiving anything of size or value for starting or finishing their study activity. This result of our study adds the insight that average procrastinators can deal with the tendency to procrastinate, if the value of what they do is clear to them.

The results show that high procrastinators set certain preconditions to start or continue a task. For these students, task aversiveness is an argument to postpone or give up a task. This finding is in line with previous research (Scher and Ferrari, 2000; Ackerman and Gross, 2005; Steel, 2007) in which task aversiveness was a predictor of procrastination behavior. The present study adds insights into how students deal with the factor of task aversion. While for students with high levels of procrastination task aversiveness is an argument not to start or to stop, for students with low and average levels of procrastination, task aversion does not automatically lead to procrastination behavior. Admittedly, low procrastinators had less task aversion, because they found everything interesting and deepened their learning. This is in line with the study by Sæle et al. (2017) showing less procrastination was associated with a strategic learning approach. This was different for average procrastinators who reported experiences of task aversion. They realized that finishing the task was important to reach their goal of becoming a teacher, which helped them finish the task. This outcome is partly in line with the study by Nordby et al. (2017), which showed that for students with a low disposition to procrastinate, environmental factors had a negligible impact and for high-level procrastinators, environmental factors facilitated and augmented procrastination. The results of the present study differ for the group of students with average levels of procrastination, because in this study environmental factors had a negligible impact on students with average levels of procrastination. The students’ drive to become a teacher makes them persevere.

The results show how self-control (Steel, 2007) among students with different levels of procrastination works. This factor plays a role at different moments in which students work on study activities. An important aspect of having self-control is *effort regulation* (Richardson et al., 2012), which refers to the capacity to persist when confronted with academic challenges, and for example, start unconditionally when a task is perceived as unattractive. The results show that high procrastinators seem not able to regulate their effort. In addition, when a task turns out to be unattractive, a difference in self-control surfaces: high procrastinators then tend to stop, while average procrastinators tend to continue completing the task, as they keep the goal of becoming a teacher in mind. Low procrastinators are able to make the task interesting to themselves. A possible explanation for these results for the different learning characteristics for students with low, average, and high levels of procrastination could be the students’ level of executive functioning (Rabin et al., 2011).

Regarding fear of failure, the results show that average procrastinators compared with the other two groups seem to suffer the most from fear of failure regarding exams. The relationship between fear of failure and procrastination behavior appeared to be more complicated than became visible in previous studies (Schouwenburg, 1992; Ferrari et al., 1995; Steel, 2007). In the present study, low procrastinators did not suffer from fear of failure and had good results whereas high procrastinators also did not suffer from fear of failure, but reported poor results for exams. The high procrastinators’ explanation for their results was that they would have had good results if they would have started studying in time. Low and high procrastinators did not doubt their competence whereas average procrastinators had doubts. This result seems to confirm Haghbin et al.’s (2012) findings that the relation between fear of failure and procrastination is moderated by the level of competence. However, in the present study this seems not the case for high procrastinators. High procrastinators’ explanation for their poor results indicates an internal attribution style for failure. This finding differs from previous research (Brownlow and Reasinger, 2000) which showed that high academic procrastinators make external attributions (to context and luck) for their successes. The explanation for their poor results seem to indicate that high procrastinators have a tendency for self-handicapping behavior, which is in line with Ferrari’s (1991) finding.

The results show that self-esteem and self-efficacy make a difference in procrastination behavior. This finding confirms previous studies showing that negative self-esteem (Ferrari, 1994) and negative self-efficacy (Wolters, 2003; Klassen et al., 2008) are related to procrastination, but in the present study, we see differences between the three groups. Low procrastinators had positive self-esteem and a positive sense of self-efficacy, and relied on this characteristic when they experienced difficulties. Average and high procrastinators had much lower self-esteem. For high procrastinators, experiencing negative thoughts or feelings was a reason to stop or not start study activities. This was the case for average procrastinators: they continued and hoped that they would accomplish the task. In addition, average procrastinators doubted their self-efficacy but thought this was no reason to get stuck in procrastination. High procrastinators seemed to have a higher sense of self-efficacy but experienced more procrastination behavior.

Looking at how students perform their study activities, the results show differences between the three groups regarding awareness and control of mental thoughts. Low procrastinators seem to be connected in the moment with their study activity and are aware of what happens in the here and now. This indicates that they are in a so-called state of *presence* (Scharmer and Senge, 2008). They seem to be connected to their capacities and rely on their strengths to complete the task, and they are determined to finish it. Average procrastinators seem to have lower levels of so-called metacognitive awareness, as defined by Flavell (1979). They are focused on completing the task and less on learning from it, except when a learning activity pertains to the profession. They then reflect on their own role as a teacher. When the results of their effort disappoint them, they cannot handle it, seem to become disconnected from their capacity to change the situation, become overwhelmed by negative thoughts, do less, and postpone the task temporarily. High procrastinators seem unable to view themselves from a metacognitive perspective. When they do not perceive the activity as useful, they experience negative feelings and judge themselves negatively. They cannot overcome the negative situation and therefore, give up and do more appealing activities outside the study activity. These results confirm previous studies showing self-awareness is necessary in order to motivate corrective behavior (Carver and Scheier, 1998) and for procrastinators, low mindfulness may be a risk factor for poor physical and emotional well-being (Sirois and Tosti, 2012).

### Limitations

The results of the present study should be interpreted against the background of several limitations. A key characteristic of most qualitative studies is that they focus on participants’ perspectives and are not intended to generalize to a broader population (Creswell, 2012). The sample comprised first-year Dutch students enrolled in an elementary teacher education program at a small teachers college. Thus, the extent to which these findings can be generalized to other programs or to other cultures is uncertain.

A second limitation is that the scores on the APSI form a continuum. Hence, our distinction of three separate groups may have created a bias. Also the number of interviewed students was low, and because students with average or low levels of academic procrastination were more willing to be interviewed than those with high levels of academic procrastination, the group sizes were not equal. The students with high levels of academic procrastination were more difficult to get in touch with and seemed to be less willing to be interviewed. Therefore, it is also conceivable that we did not interview the students with the highest procrastination problems. It is also conceivable that this serves as a behavioral measure, and the results would have been different if we had interviewed more students with high levels of academic procrastination.

A final limitation is that the interviewed students talked about their learning experiences from their own perspectives. It is not clear whether how the students talked about their regulation of learning activities always corresponds to their actual behavior.

### Future Research

With the insights of the present study in mind, it would be interesting for future intervention studies to see whether programs to overcome procrastination have different effects on students with different levels of procrastination. Future research also might show whether average procrastinators are more open to overcoming their procrastination, for example, because they have stronger ideals. Perhaps this group would benefit the most from interventions and therefore, should be targeted most in intervention programs. A question to answer in future research could also be which interventions for overcoming procrastination are more helpful for average procrastinators and which for high procrastinators. The results of the present study show differences in the metacognitive awareness and degree of presence of the students in the different groups. For future research, this difference raises the question whether students with average and high levels of procrastination can be taught to enhance their metacognitive awareness (as defined by Flavell, 1979) and their level of presence as defined by Scharmer and Senge (2008), and how this affects their levels of academic procrastination.

### Implications for Practice

Because of the important role of intrinsic motivation in dealing with procrastination behavior, it can be helpful for teacher education institutions to determine to what extent students are motivated. This could have consequences for the intake procedure that takes place, as well as for the aspects of this procedure. This study showed that it especially makes sense for average procrastinators that study activities are practice-oriented. Recognizing the relevance and practical foundation of study activities makes students understand why the study activity is important for them. A stronger connection between the theory students have to study and practice can be reached by taking practical experiences as a starting point for enhancing students’ motivation and for promoting their willingness to engage in study activities. This so-called pedagogy of realistic teacher education (Korthagen et al., 2001) might make a difference in maintaining students’ motivation and improve the attractiveness of study activities and decrease students’ procrastination.

In the present study, low procrastinators seem to be most aware of their personal strengths and were engaged in their learning activities while working on them. They are also aware of their interest and their curiosity to learn more and have positive belief in themselves. Research in positive psychology (Fredrickson and Branigan, 2005) has shown that it is possible to influence people’s beliefs about themselves by supporting awareness and the enactment of their character strengths. Character strengths can be defined as positive traits reflected in thoughts, feelings, and behaviors (Park et al., 2004) and are considered an important aspect of people’s ‘psychological capital’ (Luthans et al., 2007). Examples of character strengths are curiosity, perseverance, willpower, and hope. According to Fredrickson’s (2003; 2009) broaden-and-build theory, a focus on character strengths and positive emotions expands people’s repertoires of thoughts and actions (Fredrickson and Branigan, 2005). When students are aware of their character strengths in study situations in which the students experience negative feelings, the students can use these strengths to promote their belief in their capacities, which may help them overcome their procrastination behaviors. We implemented these ideas in a field experiment in which we trained procrastinators to overcome their procrastination (Visser et al., 2017). This field experiment showed diminishing effects on academic procrastination behavior.

Although in this article we discussed an exploratory study, the findings of this study among students with low, average, and high levels of academic procrastination deepens the insights into the process of procrastinatory factors that can influence students’ learning. Factors influencing academic procrastination do not have a linear effect but are the result of how students deal with procrastination. The present study provides insights that can lead to hopeful perspectives that more is possible than we see now in the area of academic procrastination.

## Author Contributions

LV: Ph.D.-candidate at VU University Amsterdam, Netherlands. He is the principal investigator and principal author of this article. He collected and analyzed the data, and interviewed the participants. FK and JS: supervisor and co-writer of parts of the manuscript.

## Conflict of Interest Statement

The authors declare that the research was conducted in the absence of any commercial or financial relationships that could be construed as a potential conflict of interest.
